# Clinical Utility of the Pronator Quadratus Sign: A Systematic Review and Meta-Analysis

**DOI:** 10.7759/cureus.99766

**Published:** 2025-12-21

**Authors:** Israa Kadhmawi, Rahel Rashid, Mohammed Babiker, Mohamed Elbeshbeshy, Conor Magee

**Affiliations:** 1 General Surgery, Wirral University Teaching Hospital NHS Foundation Trust, Wirral, GBR; 2 Trauma and Orthopaedics, Royal Liverpool University Hospital, Liverpool, GBR; 3 General Surgery, Arrowe Park Hospital, Wirral, GBR; 4 Trauma and Orthopaedics, Huddersfield Royal Infirmary, Huddersfield, GBR

**Keywords:** distal radius fractures, pronator quadratus, pronator quadratus fat pad sign, pronator quadratus hemorrhage, pronator quadratus sign, wrist fracture

## Abstract

The pronator quadratus (PQ) fat pad (PQFP) sign, visualised as a radiolucent stripe on lateral wrist radiographs, has long been proposed as an indirect marker of distal radius or wrist fractures. However, its diagnostic reliability has been variably reported. The objective of this review is to systematically evaluate the diagnostic accuracy and prognostic value of the PQFP sign in detecting wrist and distal radius fractures.

A systematic review and meta-analysis were performed in accordance with the Preferred Reporting Items for Systematic Reviews and Meta-Analyses (PRISMA) guidelines. Electronic databases (PubMed, Embase, Cochrane Library, Web of Science, and Scopus) were searched up to September 2025. Studies assessing the PQFP sign on plain radiographs or ultrasound, using advanced imaging or clinical follow-up as reference standards, were included. Pooled sensitivity, specificity, and risk ratios (RRs) were calculated using random-effects models. Ten studies met the inclusion criteria. Six datasets contributed to the diagnostic accuracy analysis, yielding a pooled sensitivity of 0.65 and specificity of 0.85, indicating moderate diagnostic performance with substantial inter-study heterogeneity. Five studies assessed the prognostic value of a positive PQFP sign, demonstrating a 2.44-fold increased risk of underlying fracture (RR = 2.44, 95% confidence interval (CI) 1.31-4.56, p = 0.005). Sensitivity analysis excluding ultrasound-based data maintained statistical significance (RR = 1.83, 95% CI 1.36-2.47; I^2^ = 0%).

The PQFP sign shows moderate specificity but variable sensitivity for distal radius fractures. A positive PQFP sign substantially increases the likelihood of fracture and should prompt further imaging, especially when radiographs appear equivocal. However, due to heterogeneity and modest sensitivity, the sign should not be used in isolation for fracture exclusion.

## Introduction and background

The pronator quadratus (PQ) is a small, quadrangular muscle that attaches to the anterior surfaces of the distal ulna and radius, forming a distinct anatomic compartment [1,2]. A narrow radiolucent band, known as the PQ fat pad (PQFP), lies above the muscle and serves as a boundary between it and the flexor digitorum profundus. On lateral wrist radiographs in individuals without pathology, the PQFP typically appears as either a straight line or a mildly convex stripe running parallel to the distal radius [3]. However, this overlying fat plane may appear anteriorly displaced or obliterated when the PQ muscle swells within its compartment, typically as a result of haemorrhage secondary to traumatic injury, such as a distal radius fracture [1-3]. This is referred to as “PQ sign” or “PQ fat pad sign,” which was first described by MacEwan in 1964 [4].

MacEwan reported high sensitivity and specificity values (98% and 94%, respectively); however, subsequent studies have not replicated such diagnostic accuracy for this sign. Zammit-Maempel et al. [5] found that only 51% of forearm fractures demonstrated an abnormal pronator fat pad. Similarly, Annamalai and Raby [6] reported even lower diagnostic accuracy for the PQ sign, with sensitivity and specificity values of 26% and 70%, respectively, in cases of radiographically occult fractures later confirmed on magnetic resonance imaging (MRI).

Some studies have attempted to mitigate the accuracy of the sign by measuring the PQ muscle thickness; Sasaki and Sugioka [3] considered the sign as positive if PQ thickness was more than 7 mm, while Fallahi et al. [7] considered the threshold to be 8 mm in females and 9 mm in males. However, Sato et al. [8] reported a significant difference in PQ thickness between dominant (4.7 ± 1.2 mm) and non-dominant (4.4 ± 1.1 mm) hands of healthy adults, showing that direct thickness measurement is not reliable. To account for these variations, Sun et al. investigated the PQ muscle-to-bone thickness ratio (MBR) and proposed a threshold value greater than 0.4 as an indication for MRI [9].

Owing to the variability in reported sensitivity, specificity, and measurement thresholds across studies, we undertook a systematic review and meta-analysis to synthesise the available evidence and clarify the diagnostic value of the PQ sign in distal radius injuries.

## Review

A systematic review and meta-analysis were performed in accordance with the Preferred Reporting Items for Systematic Reviews and Meta-Analyses (PRISMA) guidelines [10]. The study protocol was also registered in the PROSPERO database (CRD420251175818). The objective was to evaluate the diagnostic accuracy and clinical utility of the PQFP sign on plain radiographs for detecting distal radius or wrist fractures.

Literature review and search strategy

A comprehensive literature search was conducted on 26 September 2025 across the electronic databases PubMed/MEDLINE, Cochrane Library, Embase, Web of Science, and Scopus. The search strategy employed the following keywords and Boolean combinations: (“Pronator quadratus sign”) OR (“Pronator quadratus fat pad”) OR (“Pronator quadratus fat pad sign”) OR (“Pronator quadratus hematoma” OR “Pronator quadratus haematoma” OR “PQH”) OR (“wrist fat pad sign”) OR (“subtle wrist fracture sign”). No restrictions were applied regarding language or publication date. Additionally, manual searching was undertaken by screening PubMed’s “similar articles” section for each included study and by reviewing the reference lists of all eligible publications to identify further relevant studies.

Eligibility criteria

Studies were eligible for inclusion if they satisfied the following inclusion criteria: involved patients of any age, including both adults and children, presenting with wrist trauma or suspected distal radius fractures. The index test (intervention) was defined as the assessment of the PQFP or its related signs - described variably as the fat stripe, line, shadow, hematoma, PQH, PQFP, or PQ sign - on plain radiographs. The comparator or reference standard included the absence of the PQ fat pad sign and/or confirmation of fracture by advanced imaging modalities such as computed tomography (CT), MRI, or ultrasound.

The outcomes of interest were measures of diagnostic accuracy, including sensitivity, specificity, positive predictive value (PPV), negative predictive value (NPV), and likelihood ratios, as well as indicators of clinical utility such as detection of occult fractures, need for additional imaging, and clinical or management outcomes.

Publications such as case reports, review articles, letters, or conference abstracts were excluded, as well as any paper not satisfying the above inclusion criteria.

Screening and selection of studies

All identified records were imported into Zotero (version 7.0.26; Corporation for Digital Scholarship, Vienna, VA, US) [11] for initial duplicate removal (34 duplicates), followed by an additional 13 duplicates removed using Rayyan.ai (Rayyan Systems Inc., Cambridge, MA, US) [12], resulting in 439 unique records. All tools used within this study were free to use.

These 439 titles and abstracts were screened for relevance in Rayyan.ai, with 429 studies excluded for not meeting the inclusion criteria. The remaining 10 studies underwent full-text review, all of which were deemed eligible and included in the final analysis. A PRISMA flow diagram was prepared to illustrate the screening and selection process.

Data extraction

Data extraction was performed independently by two reviewers (IK and RR) using a standardised form. Extracted data included author, year of publication, study design, population characteristics, imaging modality, reference standard, and diagnostic performance measures (sensitivity, specificity, PPV, NPV, and accuracy). Discrepancies were resolved through discussion or consultation with a third reviewer.

Risk of bias

The methodological quality and risk of bias of the included studies were evaluated using the Quality Assessment of Diagnostic Accuracy Studies-2 (QUADAS-2) tool, assessing four domains: patient selection, index test, reference standard, and flow/timing [13].

Statistical analysis

Pooled estimates of sensitivity, specificity, and diagnostic odds ratio (DOR) were calculated using a random-effects model (DerSimonian-Laird method) to account for heterogeneity and risk of bias. Between-study heterogeneity was quantified using the I^2^ statistic. A hierarchical summary receiver operating characteristic (HSROC) curve was generated to illustrate overall diagnostic performance. Statistical analyses were performed using Cochrane’s Review Manager (RevMan) (The Cochrane Collaboration, London, England, UK) [14] and MetaDTA (version v2.1.5; University of Leicester, Leicester, United Kingdom) [15].

Results

Study Selection and Data Extraction

**Figure 1:**
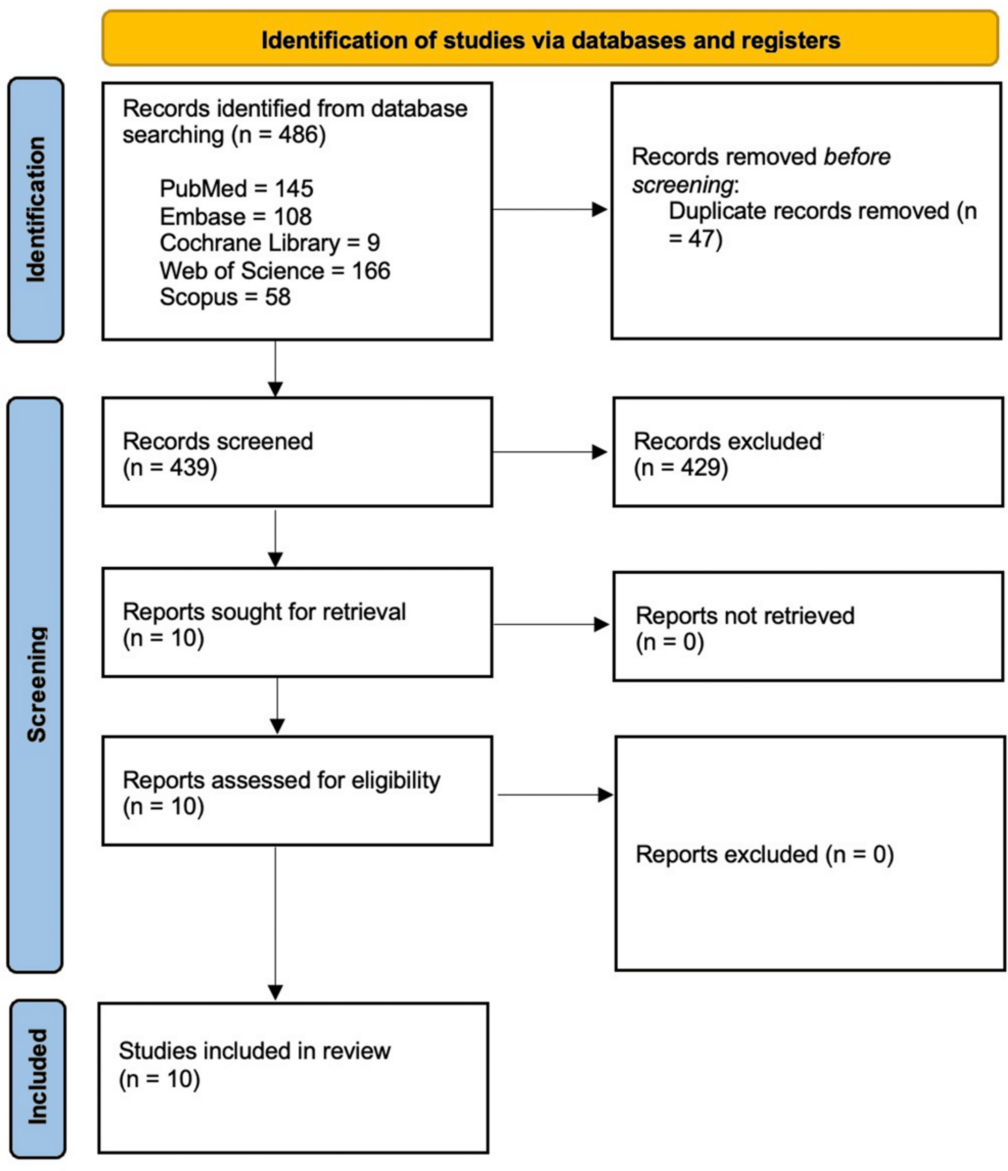
PRISMA flow diagram illustrating the study selection process. PRISMA: Preferred Reporting Items for Systematic Reviews and Meta-Analyses

A comprehensive literature search was conducted on 26 September 2025 across the following electronic databases since their inception: PubMed/MEDLINE (n = 145), Cochrane Library (n = 9), Embase (n = 108), Web of Science (n = 166), and Scopus (n = 58), yielding a total of 486 records. After removing duplicates and performing abstract and full-text review, a total of 10 studies were selected based on the inclusion criteria. Figure 1 illustrates study selection in the PRISMA flow diagram.

A total of 10 studies were included, four of which were prospective designs, and the remaining six were retrospective. Table 1 summarises the included studies and their characteristics.

**Table 1:** Summary of included studies. PQ: pronator quadratus, XR: X-ray, CT: computed tomography, MRI: magnetic resonance imaging, US: ultrasonography, NR: not reported, SD: standard deviation Credit: Calculated using Microsoft Excel (version 16.77.1) [20]

Study	Methodology	Design	Imaging Modality	Sample Size	Fracture Sample Size	Mean Age (SD)	Male Sex (%)
Loesaus et al. 2017 [16]	Retrospective	Correlating the PQ fat pad between XR and CT	XR, CT	89	44	49 (18)	54
Sun et al. 2016 [9]	Retrospective	Case-Control (normal vs. undisplaced distal forearm fractures). Measured muscle-to-bone ratio	XR	316	106	45 (19)	50
Fallahi et al. 2012 [7]	Prospective	Case-control	XR, MRI	108	68	36.5	53
Sasaki and Sugioka 1989 [3]	Retrospective	Presence of PQ sign	XR	101	42	NR	NR
Zammit-Maempel et al. 1988 [5]	Retrospective	Changes in six soft tissue planes: the pronator, dorsal radius and dorsal wrist fat stripes, the navicular, pararadial and para-ulnar fat stripes	XR	1453	439	NR	NR
Curtis et al. 1983 [17]	Retrospective	Four radiologists independently reviewed	XR	237	237	NR	NR
Annamalai and Raby 2003 [6]	Retrospective	Examining random radiographs of patients with proven MRI findings	XR, MRI	100	50	NR	NR
Sato et al. 2015 [8]	Prospective	US examination of PQ following normal radiographs	US	55	55	26.3 (22.8)	58
Snelling et al. 2022 [18]	Prospective	Clinically non-angulated forearm underwent US first, then a radiograph	US	38	38	9 years (3.4)	46
Snelling et al. 2024 [19]	Prospective	Randomised patients undergoing US	US	135	35	10.5 (3.0)	63

Risk of Bias Assessment

Risk of bias using the QUADAS-2 tool was assessed for the included studies [13]. Overall, the methodological quality of the included studies was moderate, with most studies demonstrating low risk of bias across key domains, but some concerns related to patient selection and flow and timing. Table 2 summarises the risk of bias assessment.

**Table 2:** Quality assessment of included studies using the QUADAS-2 tool. QUADAS: Quality Assessment of Diagnostic Accuracy Studies-2

Study	Patient Selection	Applicability Concerns - Patient Selection	Index Test	Applicability Concerns - Index Test	Reference Standard	Applicability Concerns - Reference Standard	Flow and Timing
Loesaus et al. 2017 [16]	Low	Unclear	Low	Low	Low	Low	High
Sun et al. 2016 [9]	Low	Unclear	Low	Low	Low	Low	High
Fallahi et al. 2012 [7]	Low	Low	High	Low	Low	Low	Low
Sasaki and Sugioka 1989 [3]	High	High	Unclear	Low	Unclear	Low	High
Zammit-Maempel et al. 1988 [5]	Low	Low	Unclear	Low	Unclear	Low	High
Curtis et al. 1983 [17]	Low	Low	Unclear	Low	Low	Low	Low
Annamalai and Raby 2003 [6]	Low	Low	Low	Low	Unclear	Low	High
Sato et al. 2015 [8]	High	Low	Unclear	Low	Unclear	Low	High
Snelling et al. 2022 [18]	High	Low	Unclear	Low	Low	Low	High
Snelling et al. 2024 [19]	Low	Low	Unclear	Low	Low	Low	High

Outcomes

Diagnostic Test Accuracy

**Figure 2:**
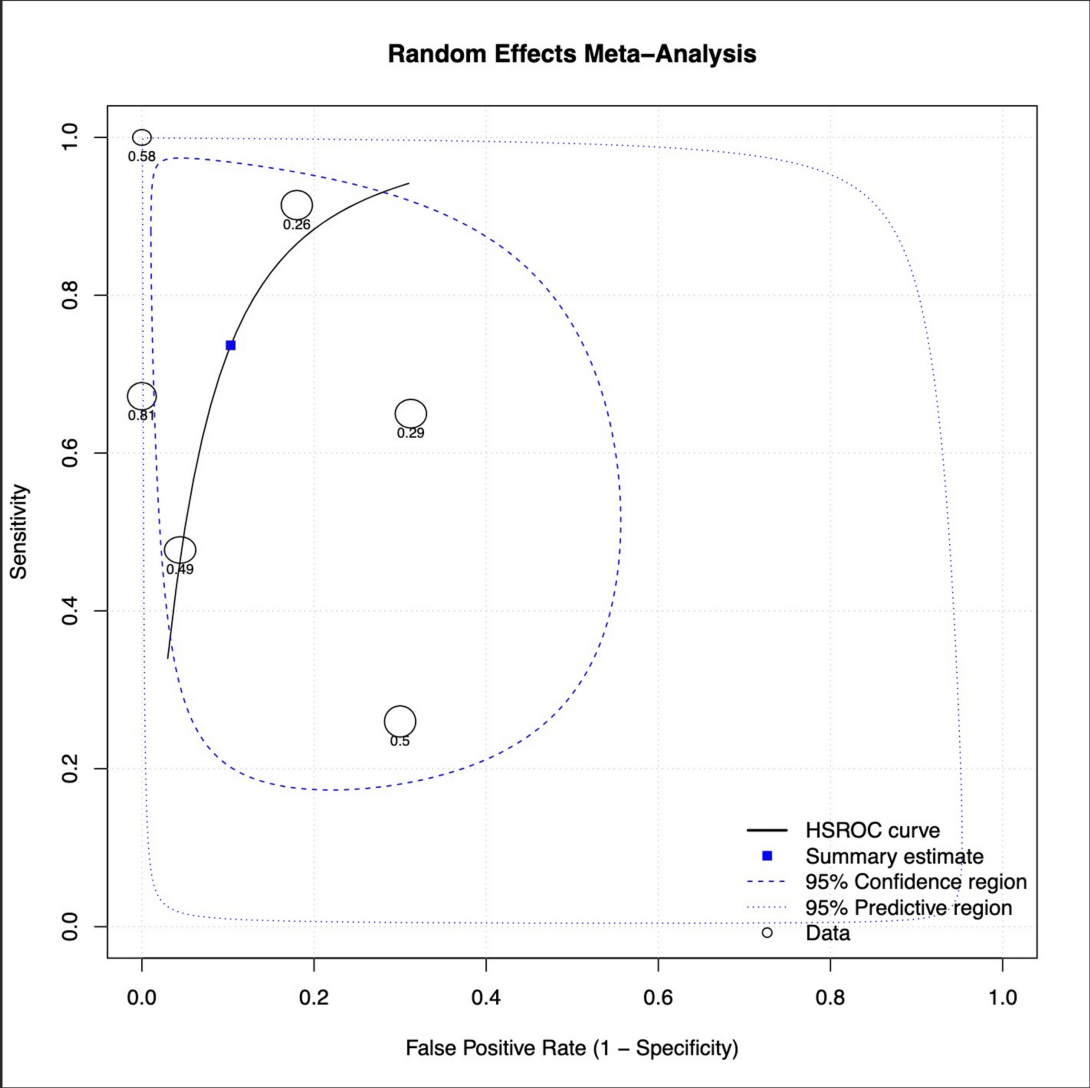
Summary receiver operating characteristic (SROC) analysis. HSROC: hierarchical summary receiver operating characteristic Credit: Generated using MetaDTA [15]

A total of six datasets were included in the diagnostic accuracy analysis. The HSROC curve from the random-effects meta-analysis was utilised [15], and this demonstrated good overall diagnostic performance (a pooled sensitivity of approximately 0.65 and a specificity of 0.85). Most individual studies clustered toward the upper-left quadrant, indicating high sensitivity and specificity. The summary estimate (blue square in Figure 2) lay near the upper segment of the curve, suggesting balanced diagnostic accuracy. The 95% confidence region was relatively narrow, indicating moderate precision in the pooled estimate, whereas the broader 95% predictive region reflected some heterogeneity between studies. Figure 2 presents the summary receiver operating characteristic (SROC) curve.

**Figure 3:**
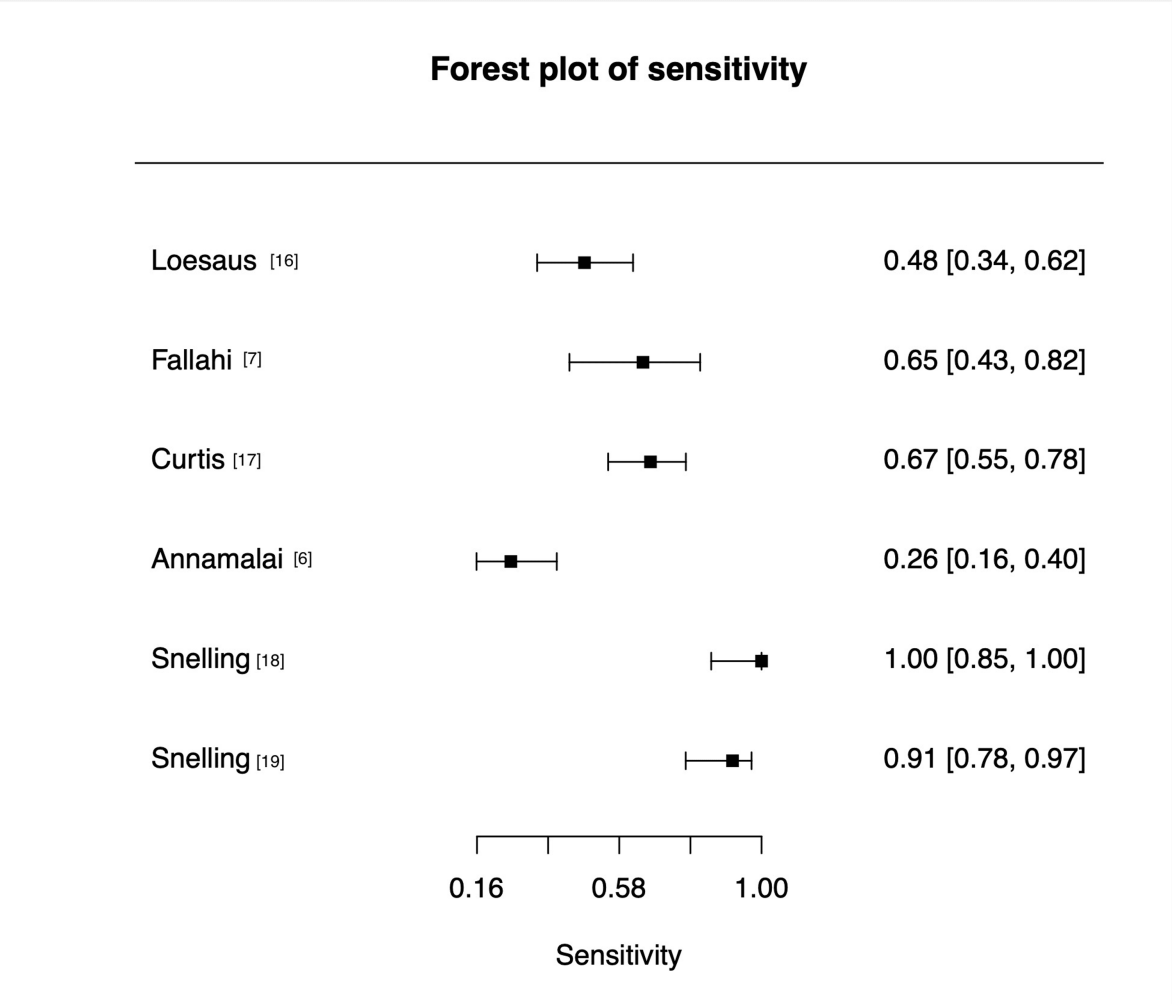
Forest plot of sensitivity estimates. References [6,7,16-19] Credit: Generated using MetaDTA [15]

Sensitivity (Figure 3) estimates demonstrated marked variability across studies, ranging from 0.26 (95% confidence interval (CI) 0.16-0.40) to 1.00 (95% CI 0.85-1.00). Four studies (Fallahi et al., Curtis et al., Snelling et al. 2022 and Snelling et al. 2024) reported moderate-to-high sensitivity (0.65-1.00), whereas Annamalai and Raby reported substantially lower performance (0.26).

**Figure 4:**
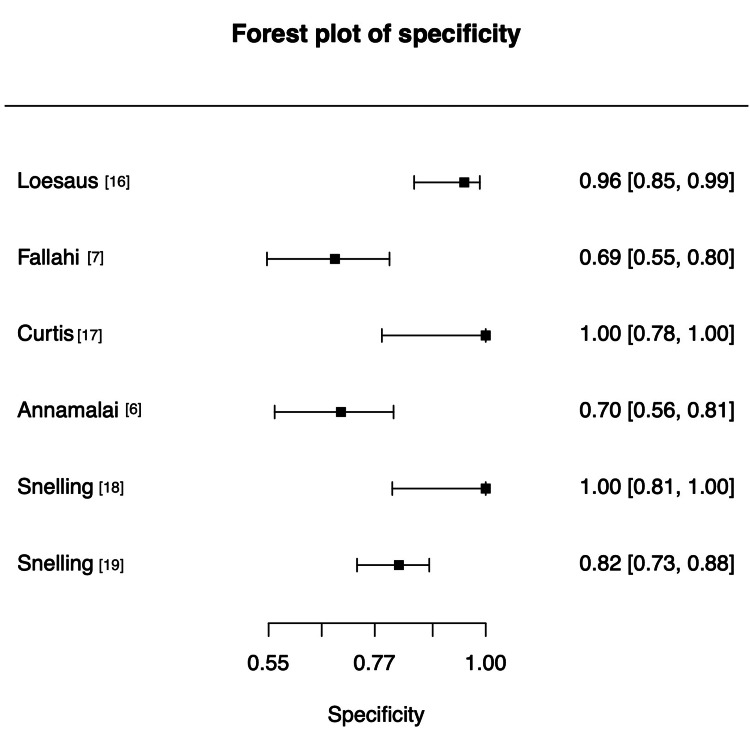
Forest plot of specificity estimates. References [6,7,16-19] Credit: Generated using MetaDTA [15]

Specificity values were consistently higher than sensitivity (as shown in Figure 4), ranging from 0.69 (95% CI 0.55-0.80) to 1.00 (95% CI 0.78-1.00). Loesaus et al., Curtis et al., and one Snelling dataset achieved near-perfect specificity (>0.95), whereas Fallahi et al. and Annamalai and Raby showed relatively lower values (≤0.70).

Prognostic Utility

Five studies assessing the prognostic value of the PQFP sign were included (Table 3), encompassing a total of 467 patients (170 with a positive PQFP sign and 297 without). Fractures occurred in 120 of 170 patients in the PQFP-positive group and in 90 of 297 in the PQFP-negative group.

**Table 3:** Studies reporting prognostic clinical data. PQFP: pronator quadratus fat pad; +: positive; -: negative; RR: relative risk; CI: confidence interval Credit: Calculated using Microsoft Excel (version 16.77.1) [20]

Study	Total PQFP+	Fractures in PQFP+	Total PQFP-	Fractures in PQFP-	RR	Reported Effect Measure
Loesaus et al. 2017 [16]	23	21	66	23	2.6	RR 2.6 (95% CI 1.84-3.73)
Fallahi et al. 2012 [7]	28	13	40	7	2.7	RR 2.64 (95% CI 1.21-5.80)
Curtis et al. 1983 [17]	41	41	34	20	1.7	RR 1.7 (95% CI 1.28-2.25)
Annamalai and Raby 2003 [6]	28	13	72	37	0.9	RR 0.9 (95% CI 0.57-1.43)
Snelling et al. 2024 [19]	50	32	85	3	18.1	RR 18.1 (95% CI 5.85-56.2)

**Figure 5:**
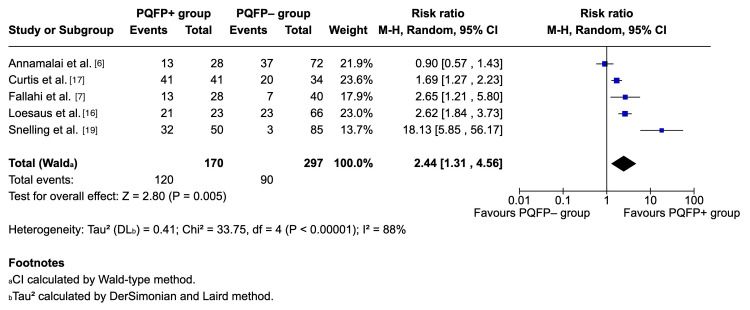
Forest plot of studies reporting prognostic clinical data. PQFP: pronator quadratus fat pad (sign); CI: confidence interval References [6,7,16,17,19] Credit: Generated using Cochrane's Review Manager (RevMan) [14]

Pooled analysis using a random-effects model demonstrated a significantly higher risk of fracture among patients with a positive PQFP sign (RR = 2.44, 95% CI 1.31-4.56, p = 0.005). Heterogeneity was substantial (I^2^ = 88%), indicating notable variability among studies. Individual study estimates ranged from RR 0.9 (95% CI 0.57-1.43) to 18.1 (95% CI 5.85-56.2). Figure 5 demonstrates the pooled analysis using Cochrane's RevMan [14].

Sensitivity Analysis

**Figure 6:**
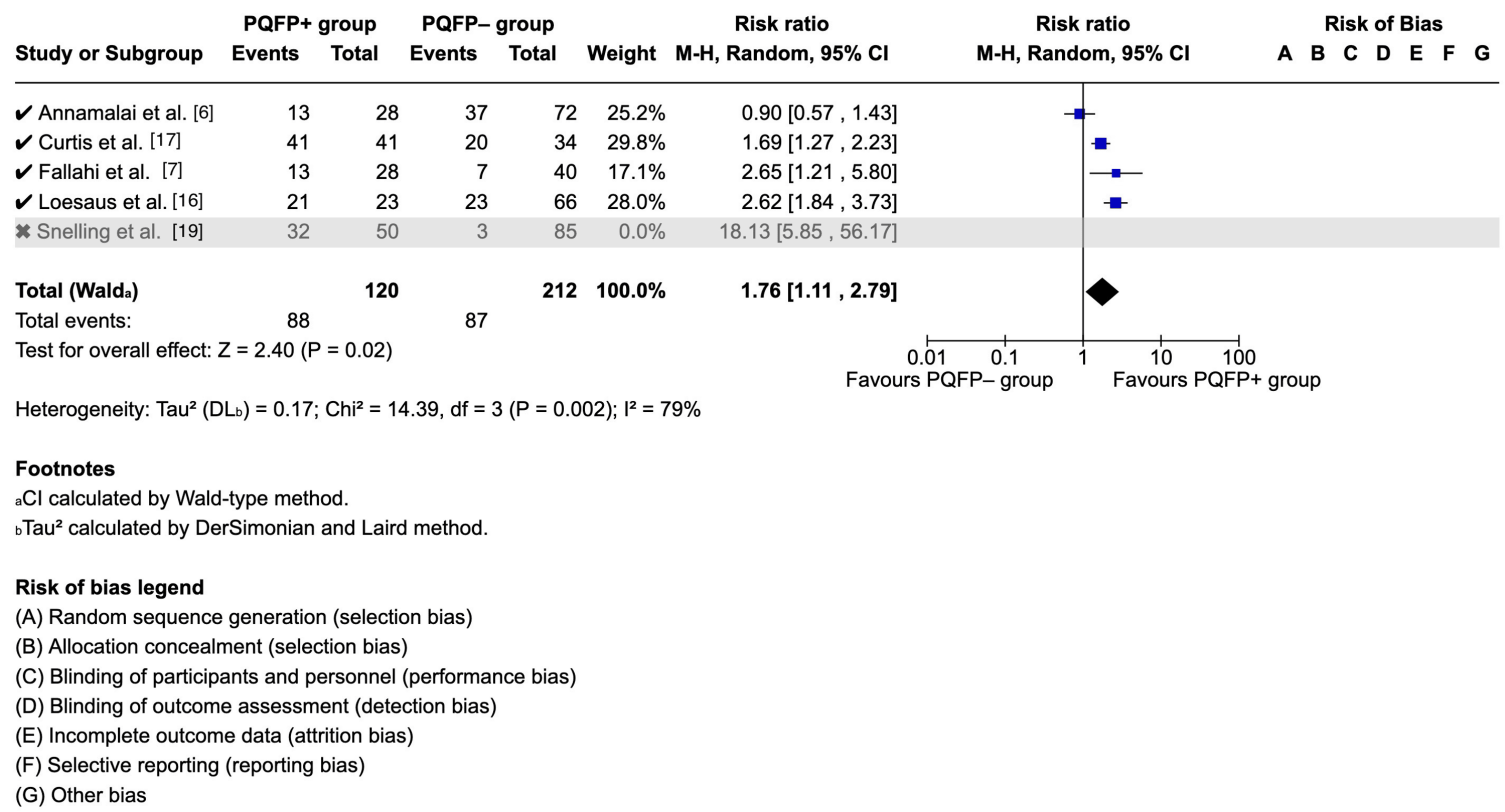
Sensitivity analysis: forest plot of studies reporting prognostic clinical data excluding ultrasound modality PQFP: pronator quadratus fat pad; CI: confidence interval References [6,7,16,17,19] Credit: Generated using Cochrane's Review Manager (RevMan) [14]

To assess the influence of methodological heterogeneity, a subgroup analysis was performed, excluding Snelling et al. (2024), the only study using ultrasound instead of radiographs. This exclusion (shown in Figure 6) reduced the pooled risk ratio to 1.83 (95% CI 1.36-2.47) with no significant heterogeneity (I^2^ = 0%). This suggests that the overall association between a positive PQFP sign and fracture risk remains significant.

Discussion

This systematic review and meta-analysis synthesised current evidence regarding the diagnostic and prognostic value of the PQFP sign in detecting distal radius and wrist fractures. The pooled sensitivity (0.65) and specificity (0.85) indicate that the PQFP sign demonstrates moderate overall diagnostic performance. Importantly, while the sign’s absence cannot reliably exclude fracture, its presence increases diagnostic confidence in the setting of trauma.

The findings corroborate early work by MacEwan [4], who first proposed the PQ sign as a fracture indicator, but they contrast with his initially reported high sensitivity and specificity (98% and 94%, respectively). More recent studies, such as those by Annamalai and Raby [6] and Zammit-Maempel et al. [5], reported substantially lower sensitivity, aligning with our pooled estimates. The wide variability likely reflects methodological differences, particularly in defining a “positive” PQFP sign, patient positioning, and the threshold for radiographic abnormality. On the other hand, false negative outcomes could be attributed to poor radiograph quality, timing of radiographs and location of the bony injury being outside the PQ muscle compartment, e.g., dorsal fractures [2,7,9]. Some studies also employed objective measurements, such as PQ muscle thickness or MBR, though these lacked standardisation and are affected by hand dominance, age, and sex [9].

The prognostic meta-analysis reinforces the sign’s clinical value: patients with a positive PQFP sign were more than twice as likely to have an underlying fracture compared to those without. This suggests that even though the PQFP sign is not diagnostic on its own, it serves as a useful adjunctive marker, particularly when radiographs appear normal but clinical suspicion remains high. The sensitivity analysis excluding ultrasound-based data (RR = 1.83, I^2^ = 0%) indicates that variability could potentially stem from differences in imaging modality rather than patient factors.

From a practical standpoint, the PQFP sign may serve as a screening clue prompting further investigation with MRI or CT, especially in cases of persistent pain or tenderness despite negative radiographs. It may also guide early immobilisation decisions pending advanced imaging. However, the sign’s low sensitivity underscores the need for a multimodal diagnostic approach, incorporating clinical findings and other radiographic indicators.

Limitations

This study is subject to several limitations. First, the included studies were heterogeneous in design, patient demographics, and imaging techniques, which limited the precision of pooled estimates. Second, several datasets were small, with wide CIs and potential publication bias favouring positive findings. Finally, most studies were conducted in adults, with limited data available in paediatric populations or specific fracture subtypes.

Despite these limitations, this review provides the most comprehensive synthesis to date on the diagnostic role of the PQFP sign and highlights the importance of standardising its definition and measurement in future research.

## Conclusions

The PQFP sign demonstrates moderate diagnostic accuracy for distal radius and wrist fractures, with high specificity but variable sensitivity. Its presence significantly increases the likelihood of an underlying fracture and should prompt further imaging when plain radiographs are inconclusive. However, due to inter-study variability and limited sensitivity, the PQFP sign should be interpreted as a supportive rather than definitive diagnostic feature. Standardised imaging criteria and larger prospective studies are warranted to clarify its clinical utility.
